# Genetic and functional characterisation of the lactococcal P335 phage-host interactions

**DOI:** 10.1186/s12864-017-3537-5

**Published:** 2017-02-10

**Authors:** Jennifer Mahony, Joana Oliveira, Barry Collins, Laurens Hanemaaijer, Gabriele Andrea Lugli, Horst Neve, Marco Ventura, Thijs R. Kouwen, Christian Cambillau, Douwe van Sinderen

**Affiliations:** 1 School of Microbiology, University College Cork, Cork, Ireland; 2 DSM Food Specialties, Delft, The Netherlands; 3 Laboratory of Probiogenomics, Department of Life Sciences, University of Parma, Parma, Italy; 4 Department of Microbiology and Biotechnology, Max Rubner-Institut, Kiel, Germany; 5 Architecture et Fonction des Macromolécules Biologiques, Aix-Marseille Université, Campus de Luminy, Marseille, France; 6 Architecture et Fonction des Macromolécules Biologiques, Centre National de la Recherche Scientifique (CNRS), Campus de Luminy, Marseille, France; 7 Alimentary Pharmabiotic Centre, University College Cork, Cork, Ireland

**Keywords:** Bacteriophage, *Lactococcus lactis*, Dairy, Fermentation, Receptor-binding protein

## Abstract

**Background:**

Despite continuous research efforts, bacterio(phages) infecting *Lactococcus lactis* starter strains persist as a major threat to dairy fermentations. The lactococcal P335 phages, which are currently classified into four sub-groups (I-IV), are the second most frequently isolated phage group in an industrial dairy context.

**Results:**

The current work describes the isolation and comparative genomic analysis of 17 novel P335 group phages. Detailed analysis of the genomic region of P335 phages encoding the so-called “baseplate”, which includes the receptor binding protein (RBP) was combined with a functional characterization of the RBP of sub-group III and IV phages. Additionally, calcium-dependence assays revealed a specific requirement for calcium by sub-group IV phages while host range analysis highlighted a higher number of strains with CWPS type A (11 of 39 strains) are infected by the P335 phages assessed in this study than those with a C (five strains), B (three of 39 strains) or unknown (one of 39 strains) CWPS type.

**Conclusions:**

These analyses revealed significant divergence among RBP sequences, apparently reflecting their unique interactions with the host and particularly for strains with a type A CWPS. The implications of the genomic architecture of lactococcal P335 phages on serving as a general model for *Siphoviridae* phages are discussed.

**Electronic supplementary material:**

The online version of this article (doi:10.1186/s12864-017-3537-5) contains supplementary material, which is available to authorized users.

## Background

Lactococcal phages present one of the most significant challenges to modern dairy fermentations. These phages are classified into ten genetically and morphologically distinct groups, of which members of the so-called 936 and P335 groups represent the most commonly encountered and problematic phages in the dairy industry [1]. The initial interaction between a lactococcal phage and its host is dictated by two factors: (a) the host-encoded target receptor molecule(s), which may be a carbohydrate, protein and/or teichoic acid component of the cell envelope, and (b) the phage-encoded receptor binding proteins (RBPs). The genetic determinant of the primary host receptor for 936 phages was discovered over a decade ago [2, 3] and this prompted further studies of host recognition and attachment by various other lactococcal phages [4–7]. Based on these studies, the primary receptor for P335, 936, 949 and P087 group phages is known to be saccharidic in nature and the variable genetic locus that is responsible for the biosynthesis of this glycan receptor or so-called cell wall polysaccharide (CWPS) has been identified and partially characterized in a number of strains. Comparative genome analysis of the variable CWPS-specifying DNA regions of six lactococcal strains permitted the identification of three CWPS-associated genotypes classified as A, B and C, and with further CWPS types predicted [8]. This study also revealed a direct correlation between 936 phage RBP genotypes on the one hand and the host-determined CWPS genotype on the other, with 936 phages displaying an apparent preference for strains that correspond to the B- and C-type CWPS.

P335 phages are a similarly frequently occurring group of phages in the dairy industry. During the past decade significant scientific attention has been directed towards the molecular and structural analysis of the P335 phages Tuc2009 and TP901-1, resulting in these phages becoming a model system for analysis of the genetic and structural characteristics of phages infecting Gram-positive bacteria [9–14]. Gene order and associated functions of lactococcal phages appears to correspond to those of coliphage lambda, a model comparator, a presumption that was proven to be justified based on functional analysis of TP901-1 [10, 15]. Recently, two P335 phages, named Q33 and BM13, isolated in North America, were shown to be genetically distinct to previously sequenced isolates [16]. Consequently, comparative genomic analysis combined with morphological analysis was employed to classify the heterogenous P335 phages, resulting in the identification of four distinct sub-groups, designated I – IV, based on distinct genetic lineages and morphological features. The latter features relate specifically to their distal tail regions as follows: sub-group I consists of just a single representative phage, BK5-T, and is typified as possessing a long fibre at the tip of its tail; sub-group II phages, representatives of which include Tuc2009, TP901-1, P335 and ul36, are endowed with a double-disc baseplate; while sub-groups III and IV represent genetically distinct phages that exhibit a small “stubby” distal tail structure.

Structural analysis of baseplates of 936 and P335 phages revealed a calcium-binding loop in the distal tail protein (Dit) of 936 phages that was absent in the P335 phages analysed at that time [11], prompting the speculation that this represents the domain required for Ca^2+^-binding in 936 phages. Many lactococcal phages have since been shown to be able to form plaques without the need for calcium, although its presence is beneficial to lysis timing and plaque size [17]. The necessity of calcium for lactococcal phage infection was the basis on which the dairy industry developed phage inhibitory medium (PIM), which was designed to limit phage proliferation by calcium limitation [18]. Despite the application of this medium, phages continue to cause considerable problems in dairy fermentations. Current knowledge on the variable requirement of lactococcal phages for calcium-dependent plaque formation explains the ineffectiveness of PIM in eradicating the phage problem [17].

The scientific goal of the current study was to assess the genetic diversity of P335 phages and their requirement for calcium in plaque formation, while also to functionally assign the RBP-encoding genes of P335 phages beyond the well-studied sub-group II phages, so as to provide unequivocal functional data for various members of this industrially important group of phages. This study is instrumental in generating a critical mass of information related to the diversity among host-recognition devices employed by Gram^+^ host-infecting phages.

## Results

### Genomic and morphological analysis of the P335 phage isolates

**Fig. 1:**
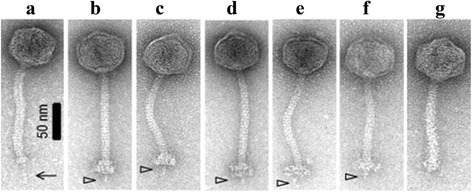
Representative electron micrographs of selected phage isolates displaying sub-group I, II or sub-group III/IV adhesion devices: (**a**) 38502 (sub-group I-like RBP); (**b**) 53801 (sub-group II adhesion module with sub-group I RBP domain); (**c**) 53802 (sub-group II phage with multicomponent baseplate); (**d**) 98104 (sub-group II phage with single component baseplate); (**e**) 98204 (sub-group II phage with single component baseplate); (**f**) 98202 (sub-group II phage with a single component baseplate); (**g**) 50902 (sub-group IV phage)

**Fig. 2:**
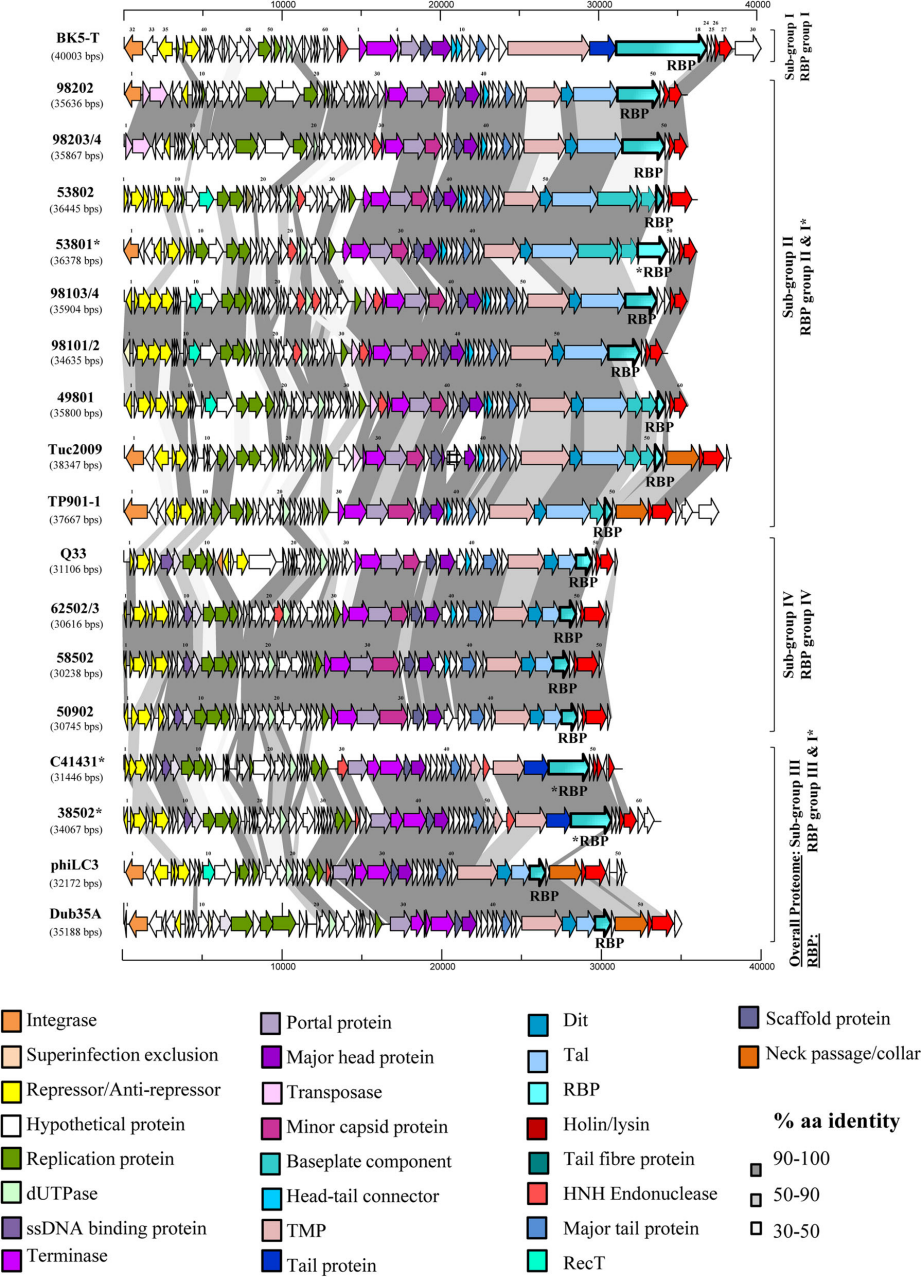
A representation of the genomes of the 17 newly sequenced phages and the closest related P335 phages among the previously sequenced phages. Very closely related phages are represented by a single representative and the related members indicated in the names on the left of the figure. Phage genomes are grouped based on overall proteome similarity while the RBP sub-group is alos indicated on the right of the figure. Where a phage exhibits overall proteome similarity to one sub-group of phages but the RBP of another sub-group, this is indicated by an asterisk in the phage names and also in the RBP indicated by text and the arrow highlighted by a bold outline. The proposed functions of the encoded proteins are colour-coded as indicated in the colour panels at the bottom of the figure

In this study, it was aimed to ascertain the genetic link to the morphological characteristics of P335 phages, in particular focusing on the tail tip region which is involved in interactions with the host. While overall nucleotide or proteomic content was widely applied to the classification of phages previously (among other tools), morphological analysis combined with sequence relatedness of the genomic region encoding the so-called adhesion device was employed as the basis for the classification of these genetically and morphologically diverse phages in this study. Table 1 outlines the characteristics of the 17 newly sequenced phages. Markov CLustering-based (MCL) alignment analysis of deduced proteomes of all currently sequenced and publicly available P335 phage genomes (27 in total, including the 17 phages described in this work) resulted in a grouping of the phages in a manner that is consistent with the previously devised system in which four sub-groups were identified (I-IV), based on overall genetic relatedness and the morphology of the tail tip region based on the complete sequence of the ten P335 genomes that were available at the time (Additional file 1: Figure S1) [16]. Furthermore, morphological analysis of the newly selected isolates also indicates that the gross morphology of the groups (based on baseplate morphology) is also maintained (Fig. 1). Phages 38502 and C41431 possess a BK5-T-like RBP, and EM images of 38502 highlight the presence of a long tail fibre protruding through the tail tip similar (though shorter) to that exhibited by BK5-T (Fig. 1a). Therefore, based on morphological similarity and sequence homologies in the region representing the adhesion module, 38502 and C41431 are assigned to sub-group I phages henceforth. Similarly, morphological analysis of phages 53801, 53802, 98104 (as a representative of 98101–4) and 98204 (as a representative of 98202–4) revealed the presence of baseplate appendages similar to those of Tuc2009 and TP901-1, and were thus considered to represent members of sub-group II phages (Fig. 1b, c, d and e). Finally, phage 50902 displays the typical small “stubby” tail tip and shorter tail that is characteristic of the sub-group III and IV phages (Fig. 1f), while comparative genomic analysis revealed sequence homology to sub-group IV members based on overall proteomic content (Fig. 2). Since Dub35A is highly identical to LC3 in the genomic region encoding the structural components, it would be expected to appear identical to this classical sub-group III phage. In summary, based on morphological characteristics and sequence homology within the adhesion module the lactococcal P335 phage sub-group I is now constituted by BK5-T, 4268, **38502** and **C41431**; sub-group II is constituted by Tuc2009, TP901-1, ul36, P335, **98204**, **98202**, **98203**, **98103**, **98102**, **98101**, **98104**, **53801**, **53802** and **49801**; sub-group III is comprised of r1t, LC3 and **Dub35A**; and sub-group IV is comprised of Q33, BM13, **50902**, **58502**, **62503** and **62502** (newly assigned members names are presented in bold-face text). A comparative analysis of the predicted protein-encoding regions of representatives of each of the four sub-groups of P335 phages is presented in Fig. 2.

**Table 1:** Characteristics of the genomes of the sequenced P335 phage isolates

Phage	*L. lactis* host	Sub-group^a^	Genome Length (kb)	No. predicted ORFs	Sequencing method	Fold coverage (approx.)	Geographical source	GC % content	Genbank accession number
C41431	SMQ-86	I	31.446	52	MiSeq	>100	Ireland	35.77	KX160219
38502	DS70385	I	34.067	60	MiSeq	667	UK	35.39	KX160204
53801	DS56538	II	36.378	53	454	498	USA	36.26	KX160207
53802	DS56538	II	36.445	58	MiSeq	552	Canada	35.97	KX160208
49801	DS68498	II	35.800	60	454	299	Germany	35.87	KX160205
98103	DS64981	II	35.904	58	454	271	Japan	35.75	KX160214
98104	DS64981	II	34.989	55	454	150	USA	36.12	KX160215
98102	DS64981	II	34.421	53	454	50	France	35.81	KX160213
98101	DS64981	II	34.635	55	454	232	UK	35.87	KX160212
98204	DS64982	II	38.334	57	MiSeq	558	UK	35.51	KX160218
98202	DS64982	II	35.636	53	454	360	Australia	36.14	KX160216
98203	DS64982	II	35.867	52	454	132	USA	36.31	KX160217
Dub35A	3107	III	35.188	53	MiSeq	>100	Ireland	35.38	KX160220
58502	DS68585	IV	30.238	50	454	37	USA	36.20	KX160209
50902	DS68509	IV	30.745	47	454	277	USA	36.04	KX160206
62502	DS63625	IV	30.617	52	454	640	UK	36.09	KX160210
62503	DS63625	IV	30.556	50	454	114	Australia	36.32	KX160211

^a^Sub-group based on sequence similarity of adhesion device module and morphological characterisation by electron microscopy

### Adhesion module analysis

**Fig. 3:**
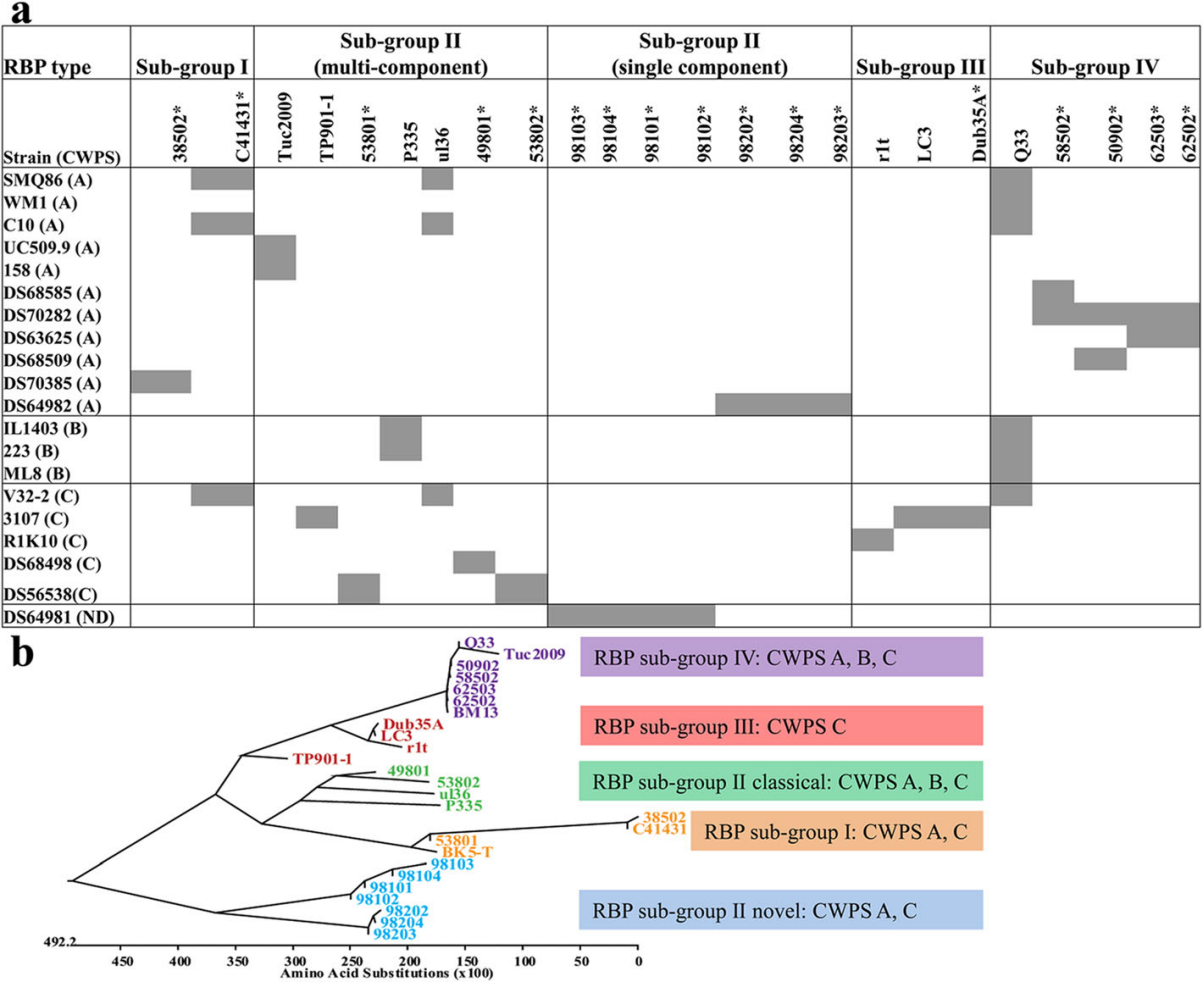
Host range of members of the lactococcal P335 phage group (Panel **a**) with sensitive strains highlighted with a *grey* box. A panel of 39 strains were employed in the host range analysis and only strains that were susceptible to infection by at least one phage within the collection are presented in Panel **a**. Phages isolated as part of this study are highlighted with an *asterisk*. The preference of the P335 phages for CWPS type A, B or C strains is presented in Panel **b** colour-coded to match the sub-groups of phage RBPs in the unrooted phylogenetic tree of the predicted RBPs of P335 phages as follows: *Yellow*: Phage RBPs with homology to that of the sub-group I phage BK5-T; *Green*: Classical sub-group II phages with homology to the namesake phage P335; *Green-Blue*: Phage RBPs with similarity to the non-classical large RBP sub-group II phages; *Red*: RBPs with homology to the sub-group III phage RBPs; *Purple*: RBPs with homology to the sub-group IV phage RBPs

**Fig. 4:**
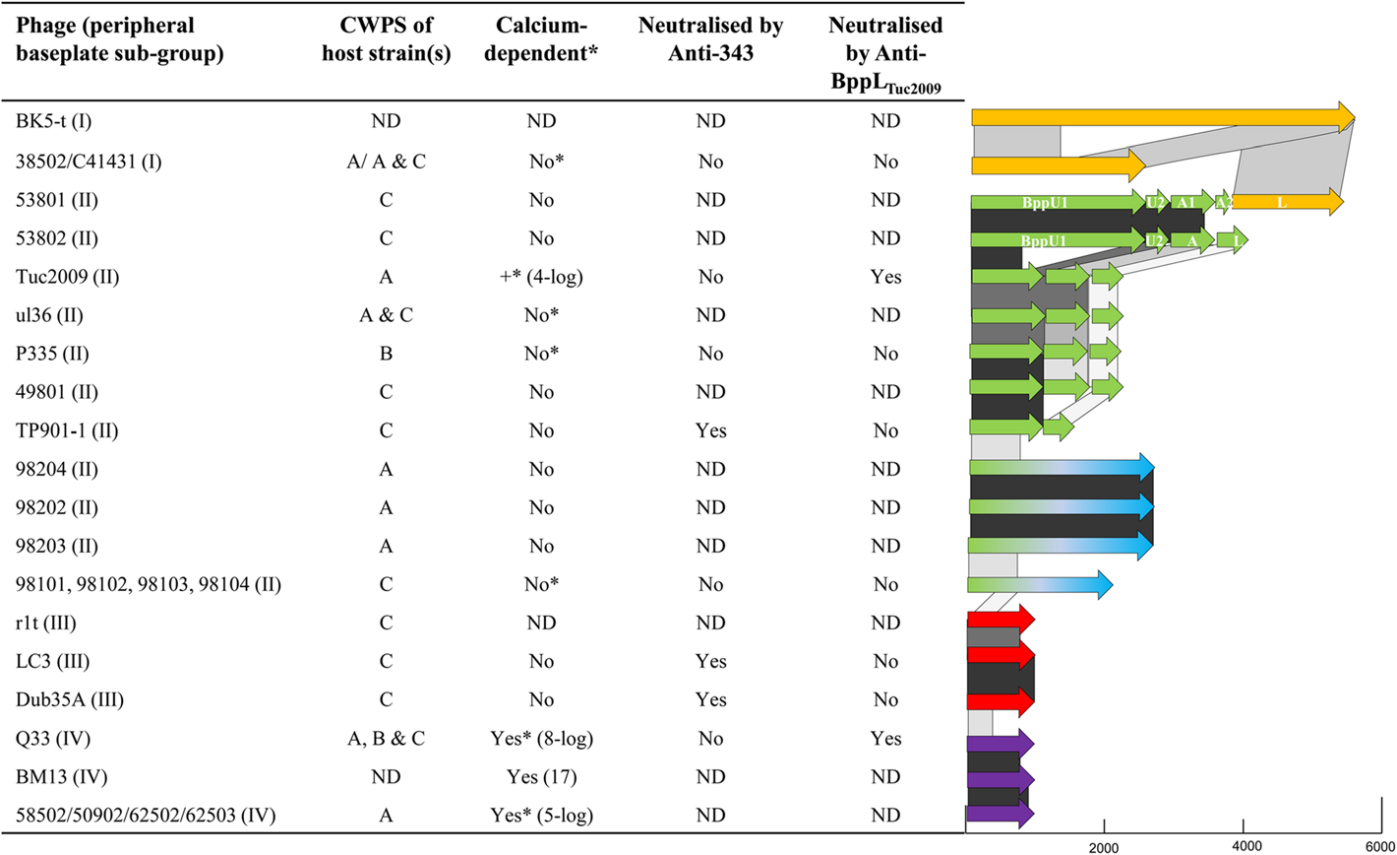
The table on the left provides an overview of the results of the calcium dependency assays and antibody neutralisation assays performed against a range of P335 phages. *Denotes that while calcium may or may not be required for plaque formation, its presence has a beneficial effect on plaque size and in the presence of EDTA a reduction in plaque size is observed. ND = Not determined. Note: The Ca-dependent characteristic of BM13 was defined in a previous study [9], while all other results presented are from data generated in this study. The *arrow diagrams* on the right are representative of the peripheral baseplate-encoding genes to indicate the single/multi-component genetic arrangements of these phages and the relatedness of the various components between the sequenced phage isolates where  indicates 95–100% aa identity;  indicates 50–95% aa identity and;  indicates 30–50% aa identity and the colour coding of the peripheral baseplate components are consistent with the RBP group colour coding in Fig. 3B. A representative scale (bp) is provided below the diagram to indicate the relative size of the ORFs encoding the various proteins of the peripheral baseplate region (BppU, BppA and BppL/RBP as appropriate)

**Fig. 5:**
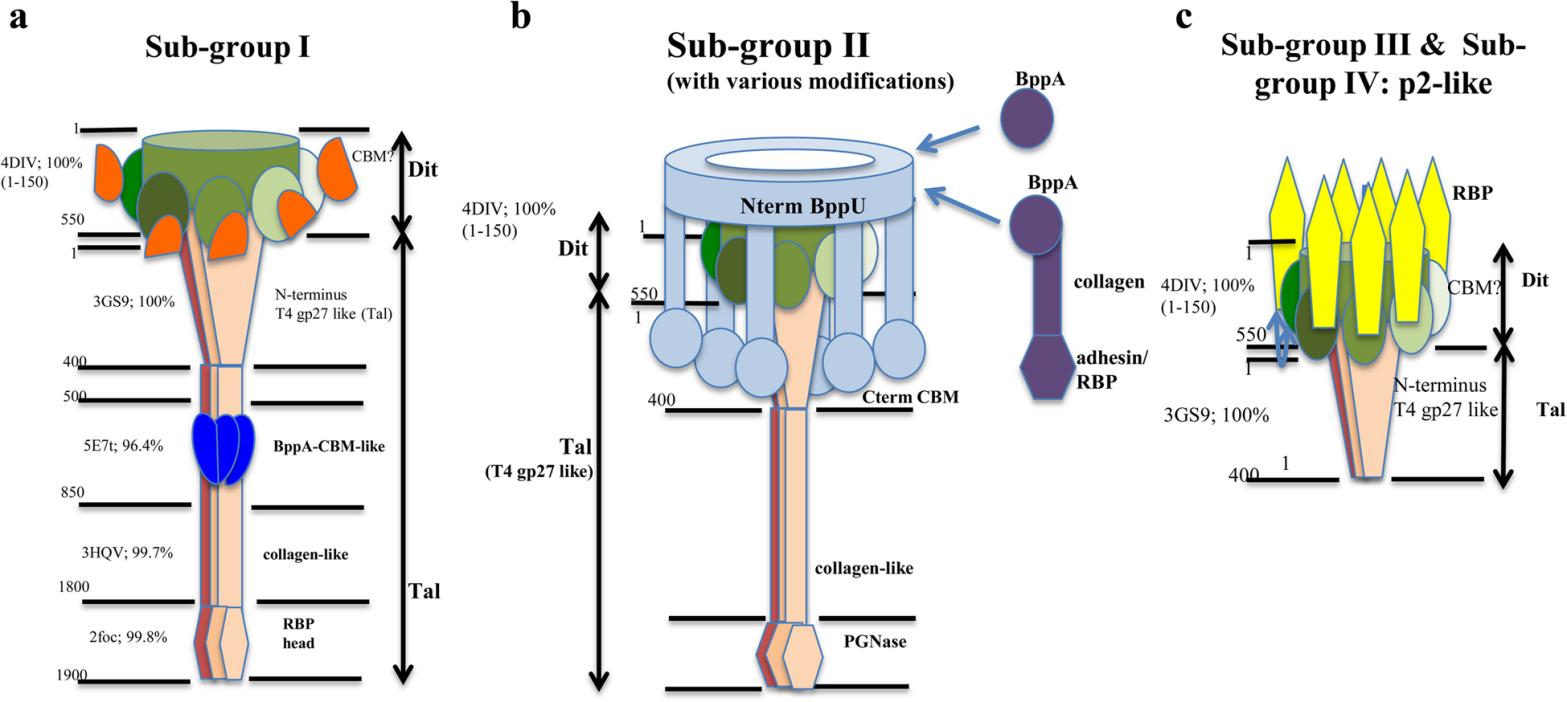
Schematic depiction of the architecture of the distal tail regions of (**a**) sub-group I, (**b**) sub-group II and (**c**) sub-group III/IV phages highlighting the domains identified by HHPred analysis

To assess the phage-derived genetic link to phage-host interactions, the adhesion modules (constituted by the genes encoding the predicted tail tape measure protein [TMP], distal tail [Dit] protein, tail-associated lysin [Tal], and receptor binding proteins [RBPs]/baseplate) harbored by the newly isolated phages were analysed. Interestingly, while the grouping of P335 phages based on overall genetic/proteomic similarity was upheld in this study, the region specifying the RBP/baseplate does not necessarily follow the same genotype grouping scheme, presumably through modular rearrangement and gene acquisition to extend or alter the host range of a given phage. Therefore, a detailed comparative analysis of the baseplate and/or RBP-encoding region of all sequenced P335 phages was undertaken (Figs. 3b and 4), while in-depth analysis of the overall adhesion modules aided in the construction of a structural model for each of the P335 phage sub-groups I, II and III/IV (Fig. 5). A detailed explanation is further provided below, dissecting the four P335 sub-groups, while Table 2 presents a summary of the identified features of the adhesion modules of the sequenced phages.

**Table 2:** List and characteristics of the baseplate ORFs of the P335 phages

	Dit	Size (aa)	Tal	Size (aa)	BppU	Size (aa)	BppA	Size (aa)	BppL/RBP	Size (aa)
Sub-group I baseplate										
BK5-T	Evolved Dit	548	Gp27 + CBM + collagen + RBP head domain	1904	none		none		none	
C41431	Evolved Dit	512	Gp27 + CBM+ head	857	none		none		none	
38502	Evolved Dit	518	Gp27 + CBM+ head	864	none		none		none	
Sub-group II baseplate										
TP901-1	Classical	253	Long PGNase	918	Classical-TP	299	none		Classical	163
Tuc2009	Classical	253	Long PGNase	906	Classical-Tuc (extended)	322	Classical	286	Classical	173
49801	Classical	253	Long PGNase	929	Classical-Tuc (extended)	322	Idem Tuc	286	Classical	175
ul36; *TP-like	Classical	253	Long PGNase	908	Classical-Tuc (extended)	322	Classical	303	Classical	165
P335; *TP-like	Classical	253	Long PGNase	918	Classical-Tuc (extended)	322	Classical	277	Classical	160
53802	Classical	253	Long PGNase	961	•BppU1:Nt + CBM + UNK •BppU2: BppU-Ct	832 107	Classical	294	TP901-like	161
53801	Classical	253	Long PGNase	961	•BppU1 idem 53802 •BppU2 : BppU-Ct	832	•BppA1: +Ig-domain •BppA2:+collagen + head	343 618	None	
98101-4; *A118-like	Classical	253	Long PGNase	929	BppU-Nterm + helix+ unknown (CBM)	672	none		None	
98202-4; *A118-like	Classical	253	Long PGNase	918	BppU-Nterm + long Linker + Cterm CBM	891	none		None	
Sub-group III baseplate										
LC3	Classical	298	Short p2-like	385	none		none		p2 like;TP head	343
Dub35A	Classical	298	Short p2-like	386	none		none		p2 like;TP head	343
r1t	Classical	298	Short p2-like	385	none		none		p2 like;TP head	349
Sub-group IV baseplate										
BM13	Classical-p2	290	Short p2-like	371	none		none		p2 like;TP head	342
Q33	Classical-p2	290	Short p2-like	371	none		none		idem	342
58502	Classical-p2	290	Short p2-like	371	none		none		idem	342
62502-3	Classical-p2	290	Short p2-like	371	none		none		idem	342
50902	Classical-p2	290	Short p2-like	371	none		none		idem	342
Other lactococcal phage sub-groups										
p2 (936 group)	Arm extd	298	Short	375	none		none			264
1358 (1358 group)	Arm extd	349	Unknown	537	none		none			393

PGNase = Peptidoglycan hydrolase; Extd = extended; Ct = C-terminus. * Indicates a similar genome architecture within the module encoding the adhesion device to that of TP901-1 (TP) or the *Listeria* phage A118

#### Sub-group I adhesion modules

Phage BK5-T is the representative model selected for analysis of the sub-group I adhesion module. This phage encodes a large Dit protein (548 aa) followed by a protein that is believed to specify both the Tal and RBP functions at its N- and C-terminus, respectively. Based on a previous morphological analysis this phage was shown to possess a very long tail fibre protruding from the distal region of the tail that is likely corresponding to this fused Tal-RBP protein [16]. While phages C41431 and 38502 group together with the sub-group III phages based on overall content by MCL analysis, they possess (Tal/)RBPs with a higher level of similarity to that of BK5-T compared to those of other representatives of sub-group III (Additional file 2: Figure S2). Furthermore, phage 53801, which groups with sub-group II phages in MCL analysis of overall proteomic content, encodes a RBP with similarity to the RBP domain of BK5-T. HHPred analysis of the Tal/RBPs of C41431 and 38502 highlight the conservation of the gp27-like Tal domain at the N-terminus (1–400) followed by a collagen helix (approx. from residues 400 to 500) and a RBP head domain (residues 730–857) (Fig. 4). The Tal/RBP of BK5-T possesses a long collagen-like fibre between a BppA-like carbohydrate binding module (CBM) and the head domain (residues ~800–1700). This baseplate structure is fully comparable to that of *Lactobacillus* phages J-1 and PL-1, which also possess large, so-called evolved and CBM-containing Dit and Tal proteins [19].

In this study, we assessed the host range of the phages displaying adhesion devices with similarity to the sub-group I phage BK5-T (which may belong to otherc content, see Fig. 2) and thereby demonstrated that phage 53801 can infect a CWPS C-type strain, phage 38502 can infect a CWPS A-type strain, while phage C41431 can infect strains of CWPS A and C-types (Fig. 3A).

#### Sub-group II adhesion modules

The sub-group II phages are the best studied P335 phages with the baseplate structure of both TP901-1 and Tuc2009 resolved (with the exception of the Tal component) [6, 11]. The primary components of the baseplates of these phages are identical with the exception that Tuc2009 encodes the accessory baseplate protein, the CBM-containing BppA, which is absent in TP901-1. Detailed analysis of the adhesion modules of this group is presented herein.

The Dit proteins of all sub-group II phages are 253 residues long (Additional file 3: Figure S3A), are considered classical Dit proteins as their fold is comparable to those of X-ray crystal structures of SPP1 [20] and TP901-1[11] (Table 2), which have been shown to form a hexameric ring with a peripheral galectin-like domain. The Tal proteins of sub-group II phages are also well conserved (Additional file 3: Figure S3B) and these are believed to form trimers located at the distal extremity of the tail tip in contact with the Dit hexamer [21]. The N-terminus of these proteins (approx. from residue 1 to 370) possesses strong structural homology to gp27 of T4 and to a type VI secretion system component, VgrG. The sub-group II Tal proteins are between 906 and 961 residues in size with highly conserved N- and C-terminal regions interjected by a variable mid-region spanning from amino acid residues 343–605 of the protein (Additional file 3: Figure S3B).

In contrast to the relative conservation of other elements of the adhesion module of sub-group II phages, the BppU proteins are quite variable with two apparent groups: the classical BppU encoded by Tuc2009, TP901-1, ul36, P335, 49801 (with a length of 299 to 323 residues) and those encoded by 53801, 53802, 98202–4 and 98101–4, which are considerably longer (Table 2, Additional file 3: Figure S3C). Phages that encode a BppA also possess a C-terminal extension in their respective BppU proteins that acts as the BppA “hanger” anchoring it to the baseplate [9]. BppUs are assembled as trimers with their N-terminal 130 residues assembled around the Dit ring. A three-helix bundle is projected out of the ring adjacent to a β-sandwich trimeric structure that serves to attach the RBP.

The majority of sub-group II phages incorporate BppA elements which add CBMs to the baseplate [6]. The N- and C-termini of the BppAs of these phages are well conserved and are interrupted by a much less conserved middle region (approx. from residues 50 to 200) corresponding to a variable CBM, suggesting phage-specific carbohydrate binding affinities (Additional file 3: Figure S3D).

The newly isolated phages including 53801, 53802, 98103 and 98202 appear to possess baseplates that are similar to those found in Tuc2009 and TP901-1, yet with some modifications, expanding the current knowledge on sub-group II phages. For example, phage 53802, which in many respects appears similar to the Tuc2009/TP901-1 arrangement, encodes a much larger BppU (termed BppU_53802(1)_, which is 832 aa in length as opposed to the Tuc2009/TP901-1 BppU which is 322 aa; Table 2). BppU_53802(1)_ corresponds to the BppU of TP901-1, but does not possess the RBP attachment domain mentioned above. Furthermore, BppU_53802(1)_ possesses a large rhamnogalacturanase A-like CBM or cazyme domain (residues 230–567). Additionally, phage 53802 encodes a second BppU-like protein (termed BppU_53802(2)_), which exhibits structural identity to a short BppU β-sandwich N-terminal domain (Table 2). Phage 53801 is similar to this but encodes two BppA-like proteins (BppA1 and BppA2). BppA1 is similar to the Tuc2009 classical BppA, while BppA2 possesses a collagen-like linker domain and a phage p2-like (a 936 group phage) RBP-like domain (residues 448–613). Phages 98103 and 98202 further diverge from the Tuc2009 sub-family as they are devoid of BppA and encode a distinct RBP, yet specify a long BppU. This highlights the diversity of arrangements of the sub-group II adhesion devices, which may incorporate a single component, large RBP-encoding gene or multi-component peripheral baseplate proteins (BppU, A and L) (Figs. 2, 3a, 4 and Additional file 3: Figure S3E).

The sub-group II phages are capable of infecting CWPS type A and/or C, or only CWPS type B strains highlighting the flexibility of interactions of this group of phages, possibly through the diversification of RBP and the presence of large, multi-domain RBP proteins with multiple carbohydrate binding domains, in many cases, observed among the sub-group II phages (Figs. 3a and 4).

#### Sub-group III adhesion modules

The Dit proteins of sub-group III phages are larger than that of TP901-1 and are, in fact, closer in size to that of the 936 group phage p2 (Table 2). In phage LC3, a 25-residue loop is incorporated within the galectin domain, resembling that of phage p2, yet not present in the TP901-1-like Dit. In congruence with the 936 phage p2 Dit similarities, the Tal of this phage consists only of the T4 gp27-like domain (Additional file 4: Figure S4A & B). Sub-group III is comprised of a small group of phages including r1t, LC3 and Dub35A. These phages display a degree of heterogeneity among their respective RBPs. The RBPs of LC3 (encoded by *orf343*_*LC3*_) and Dub35A (*orf48*_*Dub35A*_) are 94% identical at the amino acid level and both infect *L. lactis* 3107 among the strains employed in this study. The RBP encoded by r1t (*orf45*_*r1t*_) bears over 90% aa identity to that of LC3 and Dub35A across most of its protein length, yet exhibits a unique C-terminus reflecting its host-specific interacting domain likely explaining its distinctive host range.

#### Sub-group IV phage adhesion modules

Sub-group IV is embodied by Q33, BM13, 58502, 50902, 62502 and 62503. The structural characteristics of this group mirror those of the sub-group III phages with the Dit and Tal displaying the characteristics of the 936 group phage equivalents. These phages all employ a small protein of approximately 340 amino acids as their RBP and are typified by having “stubby” distal tail regions unlike the typical double-disc or broad baseplate encoded by sub-group II phages or long tail fibre structures typically observed in sub-group I phages [9]. In this regard, they appear more like the sub-group III phages r1t and LC3, which also display the stubby distal tail region and encode small RBP proteins. These RBPs are well conserved within the group and there are no component members with significant homology with P335 phage RBPs beyond this group (Additional file 4: Figure S4C). The sub-group IV phages are largely confined to infecting CWPS type A strains with the exception of Q33, which infects multiple strains of various CWPS types (Fig. 3A and 4).

### P335 phages in the collection display a preference for CWPS type A and C strains

Seventeen lactococcal P335 phages were isolated from whey samples derived from cheese production facilities across Europe and North America, Japan and Australia. The host range of the phages was assessed against the bank of 39 lactococcal strains and the results are presented in Fig. 3A. This assessment revealed unique infective profiles for each of the assessed phages, while also highlighting a narrow host range of these phages, typical of lactococcal phages, with the exception of Q33, which was previously shown to be a broad host range phage.

Strains that were identified as sensitive to the phages in this study were typed according to the multiplex PCR system devised in 2013 [22], as CWPS genotype A, B, C (or unclassified), based on the size of the generated PCR amplicon (see Methods). Using this system, it seems that the P335 phages within our collection largely infect CWPS type A strains with a slightly lower number of CWPS type C host strains and with very little preference for type B strains (Fig. 3A). Of the 39 lactococcal strains assessed in this study, 11, 3, 5 and 1 CWPS A, B, C or unknown type strains were infected by 12, 2, 10 and 4 phages, respectively. While plaque assays were used in this study to derive if any trends in infective profiles could be observed, it cannot be precluded that the phages employed in the study may bind to additional strains where plaque formation is not necessarily observed, which may alter the strain preference landscape within another phage/strain collection. Therefore, the relationship between P335 phage isolates and strain collections should be assessed on the specific collections in future studies to validate this finding.

### Calcium-dependent plaque formation by P335 phages

In previous studies, we have shown that TP901-1, ul36 and P335 do not require calcium for plaque formation, unlike members of the 936 phage group and other P335 phages, including Q33 and Tuc2009, which exhibit a calcium dose-dependent plaque formation phenotype [11]. To assess the full extent of the requirement for calcium among P335 phages and to assess a genetic linkage to this phenotype, calcium dependency assays were undertaken. In this assay, the efficiency of plaquing by phages in the presence of 0.1 mM EDTA (to chelate available divalent cations) relative to the titre in the presence of 10 mM CaCl_2_ was determined including control phages for which this data had previously been determined and are indicated below. This assay revealed that TP901-1 (control), 98202, 98203, 98204, 53801, 53802, Dub35A, LC3, ul36 (control) and P335 (control) were calcium-independent both in terms of plaque formation and size (Fig. 4). Conversely, Tuc2009 (control), Q33, (control), 58502, 62502, 62503 and 50902 display Ca-dependency with Q33 unable to produce plaques in the presence of EDTA and at least a 4-log reduction in E.O.P. for the remainder of the affected phages. Additionally, while C41431, 38502, 98101–4, ul36 and P335 do not show significant reduction in E.O.P. in the presence of EDTA, there is a very clear reduction in plaque size, which may be an indication of burst size reduction. Therefore, calcium appears to play a beneficial role in the development of these phages, even if not essential for plaque formation.

### Identification of the RBP of LC3 & Q33

The baseplates of the sub-group II phages Tuc2009 and TP901-1 are well studied and the baseplate elements have been identified and functionally characterized through structural and biological analyses [6, 9, 11, 23]. Therefore, based on similarity searches the receptor recognition functions of the sub-group II phages can be attributed to the encoded BppL (or RBP) proteins and to the C-terminal end of the large single gene RBPs of the sub-group II phages. This also aids in the positive assignment of the RBP of Q33 and LC3/Dub35A since the C-termini of their predicted RBPs bear homology to the BppL proteins encoded by Tuc2009 and TP901-1, respectively. Anti-343_LC3_ antibodies were employed in infection inhibition assays and it was observed that LC3, Dub35A and TP901-1 infection was almost completely inactivated by pre-incubation with these antibodies through binding and neutralizing the distal tail region (Fig. 4 and Additional file 5: Figure S5). This is consistent with the sequence homology observed between the proposed RBPs of these phages (94% aa identity). Furthermore, phages with non-homologous RBP sequences were not observed to be neutralized by these antibodies (Additional file 5: Figure S5). Since we were unable to obtain antibodies against the proposed RBP of Q33 (as a representative of the sub-group IV phages) and due to the sequence similarity of the C-terminus of RBP_Q33_ with BppL_Tuc2009_ (77% aa identity across the C-terminal end of the Q33 RBP), antibody inactivation assays were performed with the same set of phages using antibodies against BppL_Tuc2009_ to ascertain if infectivity of Q33, as expected, could be inhibited in a similar manner. Indeed, this resulted in a 4-log reduction in E.O.P. for Tuc2009 and a 2-log reduction in E.O.P. for Q33, while none of the other tested phages were affected by the presence of the Anti-BppL_Tuc2009_ antibodies (Fig. 4 and Additional file 5: Figure S5).

### Multiplex PCR for the classification of P335 phages

**Fig. 6:**
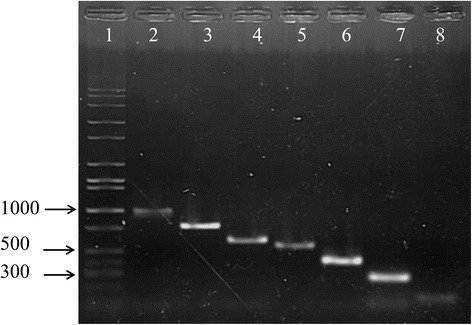
A representative image displaying the amplicons generated using individual primer pairs against the DNA extract of whey-based phage mix suspension. Lane 1: Molecular weight marker (Full scale 1 Kb ladder; Fisher Scientific); Lane 2: 38502 (sub-group I adhesion device); Lane 3: 62503 (sub-group IV); Lane 4: Tuc2009 (sub-group II, multi-component peripheral baseplate); Lane 5: 53801 (sub-group II, multi-component peripheral baseplate); Lane 6: 98204 (sub-group II, single component peripheral baseplate); Lane 7: 98104 (sub-group II, single component peripheral baseplate); Lane 8: LC3 (sub-group III). Similar results were obtained from the DNA extracts of serially diluted phage suspensions (dilutions 10^−1^, 10^−2^ and 10^−3^ but not 10^−4^). The size of representative bands on the MW marker are provided on the left

Seven pairs of primers were designed to facilitate the detection and classification of emerging P335 phage isolates. Primers were designed based on the RBP-encoding genes of phages belonging to sub-groups I, II, III or IV and owing to the apparent diversity of the sub-group II phages, primers were additionally designed to enable the detection of the various RBP-related sequences of the phages. The primers were applied as individual primer pairs and in a multiplex format using DNA extracted from phage/phage mixtures suspended in whey. When individual primer pairs were applied, all phages could be specifically detected (Fig. 6) from a mixed phage suspension with a limit of detection in whey of approximately 10^3^ pfu.ml^−1^ (data not shown); however as a multiplex PCR, only 98204 and LC3-based primers yielded the expected products and with a limit of detection of 10^5^ pfu.ml^−1^. Therefore, while these primers may not be applicable in the identification of phages in a multiplex PCR assay (likely owing to competition between primers), they may be applied to discern and classify emerging phages in surveys, which are routinely performed globally in research and industry alike.

## Discussion

Pan-genome analysis of publicly available P335 phages performed in 2014 suggested that the pan-genome of P335 phages is “open” as it appears as an exponential curve indicating that the addition of new genome sequences will increase the existing body of data for this particular phage group [24]. In the current study, 17 novel P335 phages were isolated from a wide range of geographical locations and the emergence of this additional genomic data was employed to derive a further appreciation of the genetic diversity of these phages. MCL analysis of the 27 currently available P335 phage proteomes demonstrated that the four proposed sub-groups of P335 phages are upheld [16] with newly identified members for each of the P335 sub-groups I, II, III and IV (Additional file 1: Figure S1). While the grouping of P335 phages was previously proposed based on overall genomic content and morphology, for the purposes of phage-host interaction analysis and predictions, the elements of the adhesion device were used as the differentiation factor in this study. In fact, this may be a more useful differentiation and classification tool for future isolates.

Modern fermentation facilities and starter culture providers are typically well equipped and increasingly employ molecular diagnostic tools as a means to detect and classify starter strains and their phages. Therefore, up-to-date methods are required to aid in the constant battle against phage predation. Molecular tools to classify lactococcal strains have previously been described [22], as have tools for the detection and classification of lactococcal phages [25]. However, while the phage classification scheme devised over 15 years ago reflected sequenced phages at that time and has been largely upheld, it is no longer valid for the detection of all P335 phages since the dUTPase-encoding gene is absent in the genomes of 4268 and Dub35A. As increasing sequence data emerges for the P335 phages, the extent of complexity of their genomes is becoming apparent and without a single gene that may be used as a genetic fingerprint for the P335 phages it is impossible to detect all members of this group. To counter this problem, a PCR set-up specifically designed to reflect all known members of the P335 group was devised as part of this study, providing a useful tool for the discrimination of P335 phage isolates that emerge in phage screening studies. While a multiplex PCR approach may not be applicable to discriminate P335 phages in whey samples, it is possible to employ the multiplex PCR on purified phages and their extracts (data not shown) thus advocating its potential as a classification tool after phage isolates have been selectively propagated to complement host range data.

The RBPs of the sub-group II phages TP901-1 and Tuc2009 represent the best studied RBPs of Gram-positive infecting phages [6, 9–11, 14]. The functional assignment of the RBP of the (closely related) phages in sub-group II is justified based on these studies. Unlike the sub-group II phages, the RBP-encoding genes of phages from the other sub-groups (I, III and IV) are less well studied and the assignment is mainly based on Blast similarities and/or the relative position of their encoding gene within the genome [15]. To validate these functional assignments, antibodies specific to the predicted RBP of LC3 (sub-group III; homology to Dub35A and TP901-1 RBPs) and Tuc2009 (sub-group II; with homology to the predicted RBP of the sub-group IV phage Q33) were employed in infection neutralization assays against phages encoding RBPs from sub-group I, III and IV. The anti-343_LC3_ antibodies neutralized LC3, Dub35A and TP901-1, all of which bear sequence similarity in the C-terminal receptor-interacting domain of their RBPs (Fig. 4 and Additional file 4: Figure S4). Other phages tested in this study were not inhibited in the presence of the antibody confounding their specificity and positively identifying the RBPs of LC3, Dub35A and confirming that of TP901-1. In a similar manner anti-BppL_Tuc2009_ antibodies were proven to reduce the efficiency of plaquing of Tuc2009 and Q33. The lower efficacy of the Anti-BppL_Tuc2009_ compared to that of the anti-343_LC3_ antibodies against Tuc2009 may be a reflection of the exposed regions of the purified protein against which the antibodies were raised, which may differ from its native folding thus reducing its ability to block the active binding site of the phages.

While the majority of P335 phages employed in this study display a very narrow host range, many are capable of infecting A- and C-CWPS strains within our collection. Exceptions to the narrow host specificity observation are Q33 and BM13, which exhibit an unusually broad host range and are capable of infecting strains among all three identified CWPS types (Fig. 3A) [9]. These phages may thus recognize and attach to saccharides that are common among all lactococcal CWPS structures. Conversely, phages 98101–4 specifically infect a single strain of unknown CWPS type, which may highlight the presence of a novel saccharidic receptor moiety for these phages or that the composition and/or architecture of the CWPS of this strain is quite different to those of the currently classified types (A, B and C) (Fig. 3A). It was aimed to explore a possible link between the various RBP sub-groups of the P335 phages to CWPS type of strains as was previously observed for the 936 phages [8]. No such direct correlation between specific RBP sub-groups of P335 phages and CWPS types appears to exist within the phages/strains tested but rather that there is a higher proportion of CWPS A strains infected by the phages employed in this study (11 CWPS A strains versus 10 CWPS C, 3 CWPS B). While the current study employs a limited number of strains and phages and the results may be specific to our collection, it would appear that there is preference for CWPS type A strains (based on infection assays) among the P335 phages in this and previous studies and with a secondary preference for CWPS type C strains in contrast to the 936 phages. Should this trend hold true for P335 phage isolates that emerge in other studies in the future, it would be advisable to promote the limited application of CWPS type A (at least temporarily) in fermentation plants with a prevalence of P335 phages and potentially the incorporation of CWPS type B strains in such facilities.

Through this and previous studies, it has been established that the majority of lactococcal phages do not strictly require calcium for plaque formation although in many cases it has a beneficial effect on plaque size and the timing of lysis [17]. Here, it is observed that all sequenced sub-group IV members specifically require calcium for plaque formation and in the presence of EDTA (which chelates divalent cations), these phages display at least a 5-log reduction in E.O.P. (Fig. 4). The observation of calcium-dependent plaque formation for sub-group IV but not sub-group III phages suggests that while the sub-group IV phages may require baseplate activation like the 936 phages, sub-group III phages may be activated by an alternative mechanism or factor than calcium binding or may be in an “infection-ready” configuration thus not requiring activation. The sub-group II phage Tuc2009 is an exception among the sub-group II phages in its requirement for divalent cations for plaque-formation, which may be explained, at least partially, by the sequence homology between the C-terminus of its RBP and that of the sub-group IV phage Q33.

Bioinformatic analysis of phages infecting diverse bacterial species including *Staphylococcus aureus* phage 11, *Listeria* phage A118 and *Bacillus subtilis* phage SPP1 has revealed the conservation of structural, genetic and functional characteristics (Mahony et al., unpublished data). Furthermore, it was identified that BppA-like CBDs are widespread among phages highlighting the conservation of beneficial features among diverse phages. The architectural and modular conservation of the adhesion modules of phages infecting pathogenic and non-pathogenic Gram positive bacteria highlights the broader significance of model lactococcal phages. It highlights the conservation of genetic and structural features throughout the phage realm. It is also clear that modular shuffling and duplication among phages are common occurrences, which serve to enhance the adaptive agility and evolutionary success of these viruses.

## Conclusions

In conclusion, this study has enhanced current understanding of the host preference of P335 phages and the genetic complexity of this industrially significant group of phages. The positive identification of the RBP-encoding genes of Q33, LC3 and Dub35A serves to strengthen the functional assignment of RBPs of emerging phage isolates and consolidates lactococcal phages as the model system for *Siphoviridae* phages infecting Gram-positive bacteria with implications for food fermentations, phage evolution and phage therapeutic systems.

## Methods

### Bacteria and phages

Lactococcal host strains were grown without agitation at 30 °C in M17 broth (Oxoid Ltd., Hampshire, England) supplemented with 0.5% glucose (GM17) or lactose (LM17) and 5 μg.ml^−1^ chloramphenicol, where relevant. Phages were propagated on the appropriate *L. lactis* indicator strains, which had been grown to an approximate optical density (OD_600nm_) of 0.15 in 10 ml GM17 broth. Calcium chloride was added to a final concentration of 10 mM prior to infection of the culture with approximately 10^7^ plaque forming units (pfu) or a single plaque of the relevant phage and incubated at 30 °C or room temperature until lysis had occurred. The lysates were filtered through a 0.45 μm filter to remove any residual bacterial debris and stored at 4 °C. Plaque assays were performed using the previously described double agar method [26]. This method was also applied for host range analysis performed against a set of 39 *L. lactis* industrial starter and laboratory reference strains (sensitive strains only listed in Fig. 3A).

### Phage screening

Whey samples from cheese production facilities in Europe, USA, Canada, Australia and Japan were screened against the above-mentioned bank of 39 strains and individual plaque isolates were propagated on the identified sensitive hosts. The individual isolates were plaque purified twice and propagated on the primary host strain to a titre of at least 10^7^ pfu.ml^−1^ and filter sterilized to remove remaining bacterial debris. These lysates were subsequently employed in host range analysis of the isolated phages. Consequently, 17 phage isolates were identified and selected for genome sequence analysis, the characteristics of which are outlined in Table 1.

### DNA preparation, genome sequencing, assembly & annotation

DNA for sequencing of the 17 P335 lactococcal viruses listed in Table 1 was extracted as previously described [7, 16]. For genome sequencing 5 μg of extracted DNA was used as verified by nanodrop quantification. Confirmatory molecular ID tests were also conducted on the DNA extract prior to shipment to the contract sequencing facility (MiSeq: GenProbio, Parma, Italy or 454 FLX: Macrogen Inc., Soeul, Korea). The MIRA software program (version 4.0.2) [27] was used for *de novo* assembly of MiSeq derived phage genome sequences to generate a consensus sequence. The individual sequence files generated by the 454 FLX instrument were assembled with GSassembler (454 Lifesciences, Branford, CT, USA) to generate a consensus sequence. Quality improvement of the genome sequences involved customized Sanger sequencing (Eurofins, Germany) of PCR products across the genomes of the phages to ensure correct assembly, double stranding and the resolution of any remaining base-conflicts occurring within homopolymeric tracts. Protein-encoding open reading frames (ORFs) were predicted using a combination of the methods Prodigal v2.6 [28] and BLASTX [29] followed by manual assessment, curation and correction of the predicted open reading frames. A functional annotation of ORFs was performed on the basis of BlastP [30] analysis against the non-redundant protein database (nr) provided by the National Centre for Biotechnology Information (located at: http://blast.ncbi.nlm.nih.gov/Blast.cgi). The proposed functions of many ORFs were further validated by querying protein domain databases Pfam [31], the NCBI Conserved Domain Database [32], and by performing homology prediction searches using HHPred [33]. The genomes were scanned for the presence of potential tRNA genes using tRNA scan SE [21]. Genome extremities were identified based on comparison to previously sequenced P335 phages.

### GenBank accession numbers

GenBank accession numbers for the previously sequenced phages are as follows: Tuc2009 (NC_002703.1); TP901-1 (NC_002747.1); LC3 (NC_005822.1); P335 (DQ838728.1); ul36 (NC_004066.1); Q33 (JX564242.1); BK5-T (NC_002796.1); r1t (NC_004302.1); BM13 (NC_021861.1).

### Comparative genomic analysis

The genomes of phages were compared using nucleotide BLAST analysis of the entire genomes and comparison of the encoded individual proteins was performed by all-against-all, bi-directional BLAST alignment [34] with an alignment (or E-value) cut-off value of 0.0001 and greater than 50% identity across at least 50% of the amino acid sequence. To identify the closest relatives of the sequenced phages and to establish the phylogeny of the phages based on their overall proteomic content, the Markov Clustering (MCL) algorithm was executed via the mclblastline pipeline v12-0678 as described previously [35]. The proposed adhesion devices were analysed using HHPred [36, 37]. The regions encoding the proposed adhesion devices are those genes encoding the tail tape measure protein (TMP) through to (but not including) the lysis cassette (holin and lysin) as they encode functions predicted to be associated with either the so-called “initiation complex” and/or “baseplate” [6, 11, 38, 39]. The characteristics of the features associated with the adhesion devices are presented in Table 2.

### CWPS-typing of lactococcal strains by multiplex PCR

Classification of the lactococcal strains employed in this study was achieved by means of a previously described multiplex PCR approach [22]. Lactococcal strains have been sub-grouped based on the operon that specifies the biosynthetic machinery for the cell wall polysaccharide (CWPS), which acts as the receptor material for many lactococcal phages [3–5, 22, 40, 41]. Three known CWPS types have been identified to date (type A, B and C), while additional, as yet uncharacterized genotypes are known to exist [22]. Primers based on unique regions specific to the CWPS type A, B and C genotypes, as well as a positive control (based on the *rmlB* gene which is conserved in all known lactococcal CWPS gene clusters) were employed to yield products of 442 bp (CWPS type A), 183 bp (CWPS type B) or 686 bp (CWPS type C) and the positive control product of 891 bp. The PCR was run under the following conditions: 95 °C for 6 min followed by 31 cycles of 95 °C for 15 s, 57 °C for 30 s and 72 °C for 1 min, followed by a final extension step at 72 °C for 7 min.

### Protein expression, purification and antibody generation

It has previously been suggested based on genomic synteny that the receptor binding protein (RBP) of LC3 is encoded by *orf343*_*LC3*_ while that of Q33 is believed to be encoded by *orf48*_*Q33* [16]_. To validate this functional prediction, we first aimed to produce antibodies specific to each of these proteins and then assessed the ability of the generated antibodies to neutralize phage infectivity. To this end two constructs were prepared each harbouring one of the proposed RBP-encoding genes for the purposes of putative RBP over-expression and purification. Primers used for cloning were purchased from Eurofins MWG (Germany). KOD high-fidelity DNA polymerase (Novagen, UK) was used for PCR amplifications. For cloning reactions, restriction enzymes were supplied by Roche (Germany) and ligations were performed with T4 DNA ligase (Promega, USA). The *orf343*_*LC3*_ and *orf48*_*Q33*_ genes were individually cloned into the BamHI and XbaI sites of pTX8048 which incorporates a hexahistidine-tag and a thioredoxin tag at the N-terminus of the encoded protein [42]. ORF343_LC3_ was expressed and purified as outlined previously [42]. Rabbit antibody production was carried out by Harlan laboratories (Leicester, UK.). Immunization was initially carried out with individual proteins supplemented with Freund’s adjuvant at a concentration of ~200 μg/ml; this was followed by five booster injections over the 112 day protocol. ORF48_Q33_ exhibited very poor expression despite considerable optimisation efforts and was therefore not amenable to antibody production.

### Antibody neutralisation assays

To determine if the anti-ORF343_LC3_ and anti-BppL_Tuc2009_ antibodies [14] are capable of inhibiting infection via RBP-blocking, in support of their suspected and proven (respectively) host interacting function, and their location at the distal region of the tail, antibody neutralisation assays were performed. These were performed by mixing 0.1 ml phage lysate (typical titres of 10^7^- 10^8^ pfu.ml^−1^) with 0.1 ml of (approximately 20 μg.ml^−1^ total protein) anti-ORF343_LC3_ or anti-BppL_Tuc2009_ antibodies, pre-bleed serum (negative control) or water (negative control). The mixtures were incubated at 30 °C for 60 min after which plaque assays were performed to establish the number of infective particles. Antibody-mediated neutralisation assays were performed against a selection of the phages employed in this study as representatives of each RBP type i.e. C41431 (representative of the sub-group I BK5-T-like RBPs); Tuc2009, TP901-1, P335 and 98103 (representative of the sub-group II RBPs); LC3 and Dub35A (representatives of the LC3-like sub-group III RBPs) and Q33 (representative of the sub-group IV RBPs). All assays were performed at least in triplicate.

### Calcium dependence determination

To determine if phages are dependent on calcium for successful infection of its host, plaque assays were performed in the presence of calcium (10 mM Ca^2+^) or in the presence of 0.1 mM EDTA to chelate divalent cations that may be available in the medium. Plaque assays were performed using an adapted version of the previously described double-agar method [26]. The relevant level of calcium or EDTA was added to both the M17 solid and semi-solid agar supplemented with 0.5% glucose and 0.5% glycine, and plates were incubated overnight at 30 °C. Plaques were enumerated and recorded as number of plaque forming units per ml of phage lysate (pfu.ml^−1^). This assay was performed using phages C41431, Dub35A, LC3, 98103, 98102, 98101, 98104, 98202, 98204, 98203, 49801, 50902, 58502, 62503, 62502, 53801, 53802 and 38502 as their requirement for calcium is currently unknown while Q33 (calcium dependent), Tuc2009 (calcium dependent) and TP901-1 (calcium independent) were also included as controls as these have previously been analysed with respect to their calcium requirements [11, 17].

### PCR for the classification of P335 phages

A multiplex PCR system was devised based on the RBP or baseplate-encoding elements of 38502, 53801, 98101, 98204, Tuc2009 (BppU-encoding gene), LC3 and 62503 as representatives of the four sub-groups of P335 phages and the corresponding distinct RBP and baseplate types. The following primer pairs were used: 62503for gaccgtgaatatagttctgatgaat and 62503Rev gtagtaattccgatttcccattctc (784 bp product); 98101for gacagaacattttataacactatccac and 98101rev caaactgtaacgcattatcactggc (268 bp product); 53801for caaagatgggaagatagagagta and 53801rev cctcgtggtgcgccggt (554 bp product); 98204for gcctttggagctttttcggttgat and 98204rev gcgccgttaggtatattatcc (412 bp product); LC3for cgttgaagtaaatggaagcttaac and LC3rev gaggatatttccccaccaattg (128 bp product); 38502for gatagtgaatctccgggagcg and 38502rev atcactgattgttactgtgtcccc (1002 bp product); and Tuc2009Ufor catgcggatgtcaatagtcaagcc and Tuc2009URev gtatcaaatccattcgcttcggttc (for the detection of BppU-encoding elements that may detect sub-group II phages with unique BppL regions). 0.1 ml of each phage (10^7^ pfu.ml^−1^) was mixed together and to the mixture 4.3 ml of whey (produced through the acidification of milk with lactic acid) was added. The total DNA of the phage mixture was extracted using the method mentioned above. The PCRs were performed using either the full set of primers (seven pairs of primers) or as individual primer pairs. The phage mixture in whey was also serially diluted four times in whey and the DNA extracted as described before. The DNA extracts of these samples were employed to assess the limit of detection of the assay. The PCR cycle employed was as follows: 95 °C × 10 min followed by 35 cycles of 95 °C × 15 s; 51 °C × 30 s; 72 °C × 1.5 min and a final extension step of 72 °C × 7 mins. Amplicons were applied to a 1.5% agarose gel and run at 100 kV for 40 min and visualised using a UV transilluminator. Validation assays were also performed in which the above-mentioned produced whey sample in the absence of deliberately added phages was assessed for background phages and was shown to be negative for amplicons (data not shown).

### Electron microscopy

Purification of phages by CsCl gradient was performed as previously described [43]. Adsorption of CsCl-purified phages to freshly prepared carbon film floated from a freshly coated mica sheet and negative staining with 2% (w/v) uranyl acetate were done as described previously [44]. The film was picked up with a 400-mesh copper grid (Agar Scientific, Essex, UK), and specimens were examined with a Tecnai 10 transmission electron microscope (FEI, Eindhoven, The Netherlands) operated at an acceleration voltage of 80 kV.

## Additional files

Additional file 1: Figure S1. Heat map of the P335 phage proteomes indicating the presence of absence of specific protein families within the replication, morphogeneis and lysis modules, respectively. The phages are grouped and colour-coded by sub-group with sub-group I phage BK5-T highlighted in blue; sub-group II phages highlighted in green; sub-group III phages highlighted in yellow and sub-group IV phages highlighted in red based on overall proteomic content. This was used to identify the closest relatives of each phage and thus in the construction of the comparative genomic figure (Fig. 2). (TIFF 1116 kb)
Additional file 2: Figure S2. Unrooted phylogenetic tree of the Dit (Panel A) and Tal/RBP (Panel B) protein sequences of the phages possessing a sub-group I RBP: BK5-T, 38502 and C41431. (TIFF 505 kb)
Additional file 3: Figure S3. Unrooted phylogenetic tree of the Dit (Panel A), Tal (Panel B), BppU (Panel C), BppA (Panel D) and RBP (Panel E) proteins of the sub-group II phages. (TIF 1257 kb)
Additional file 4: Figure S4. Unrooted phylogenetic tree of the Dit (Panel A), Tal (Panel B) and RBP (Panel C) proteins of the sub-group III and sub-group IV phages. (TIFF 593 kb)
Additional file 5: Figure S5. Bar chart representing the titre of phages tested in antibody neutralisation assays. The blue bar represents a control in which water was included in place of antibodies to assess the control titre; the red bars represent a control in which pre-bleed serum was added in place of the antibodies to reflect the effect of serum on the phages; the green bars represent the effect of incubation of anti-343_LC3_ on the plaquing ability of the assessed phages and; the purple bars represent the effect of incubation of anti-BppL_Tuc2009_ on the plaquing ability of the tested phages. Representatives of each of the four P335 phage sub-groups were selected for this analysis with C41431 used as the representative of the sub-group I since its predicted RBP bears homology to that of the sub-group I phage BK5-T. All data represented are the average of at least three independent assays. (TIFF 453 kb)

## Abbreviations

Bpp: Baseplate protein (A: accessory
CBM: Carbohydrate binding module
CWPS: Cell wall polysaccharide
Dit: Distal tail
GM17: M17 supplemented with glucose
L: Lower
LM17: M17 supplemented with lactose
MCL: Markov clustering
OD: Optical density
ORF: Open reading frame
PFU: Plaque forming units
PIM: Phage inhibitory medium
RBP: Receptor binding protein
Tal: Tail-associated lysin
TMP: Tail tape measure protein
U: Upper
