# Supplementary figures and images for: Genetic and functional characterisation of the lactococcal P335 phage-host interactions
Source: BMC Genomics. 2017 Feb 10;18:146. doi: 10.1186/s12864-017-3537-5 (PMC5301393; doi:10.1186/s12864-017-3537-5)

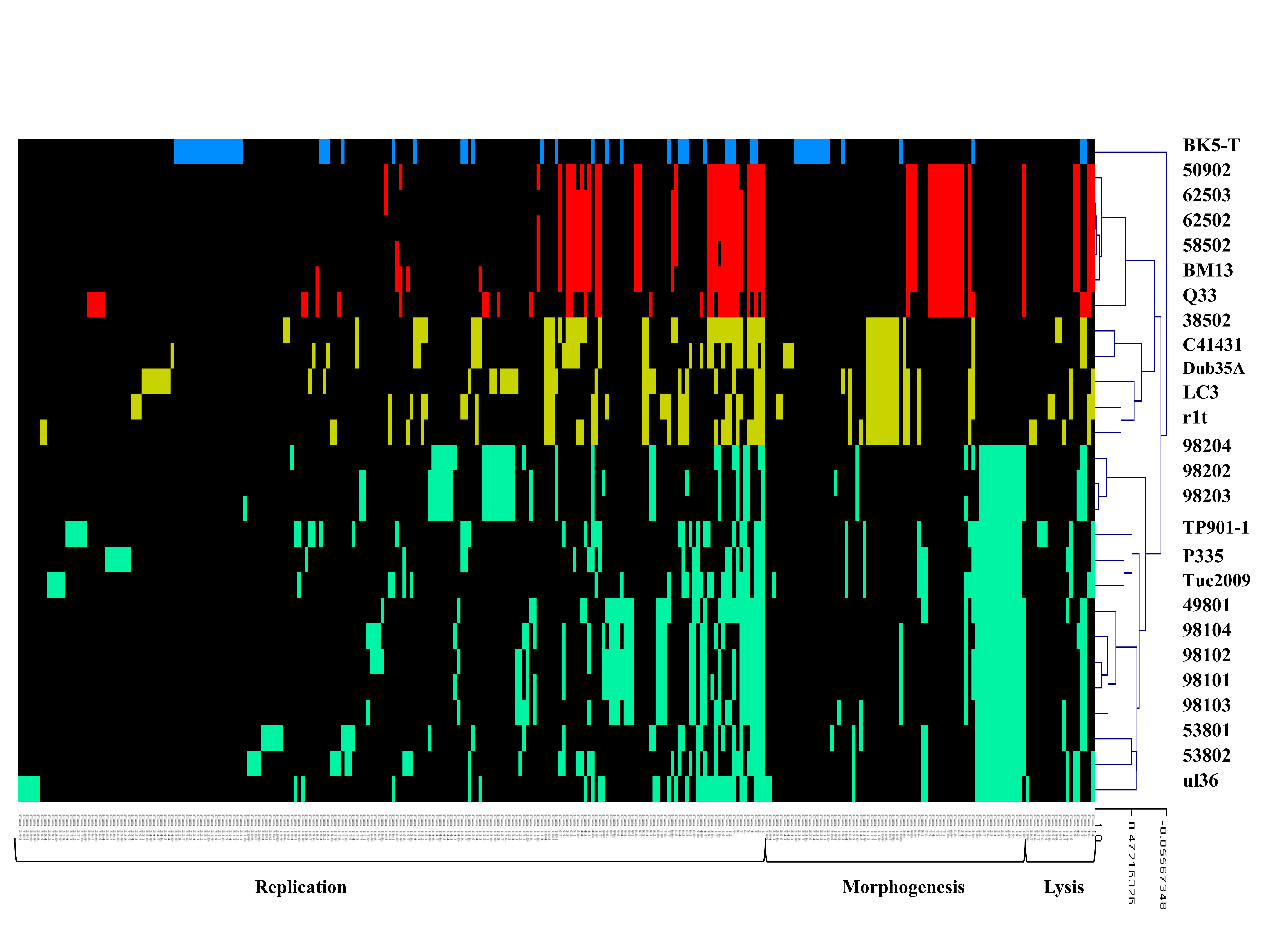

Supplement: Additional file 1: Figure S1. — Heat map of the P335 phage proteomes indicating the presence of absence of specific protein families within the replication, morphogeneis and lysis modules, respectively. The phages are grouped and colour-coded by sub-group with sub-group I phage BK5-T highlighted in blue; sub-group II phages highlighted in green; sub-group III phages highlighted in yellow and sub-group IV phages highlighted in red based on overall proteomic content. This was used to identify the closest relatives of each phage and thus in the construction of the comparative genomic figure (Fig. 2). (TIFF 1116 kb) [file 12864_2017_3537_MOESM1_ESM.tiff]

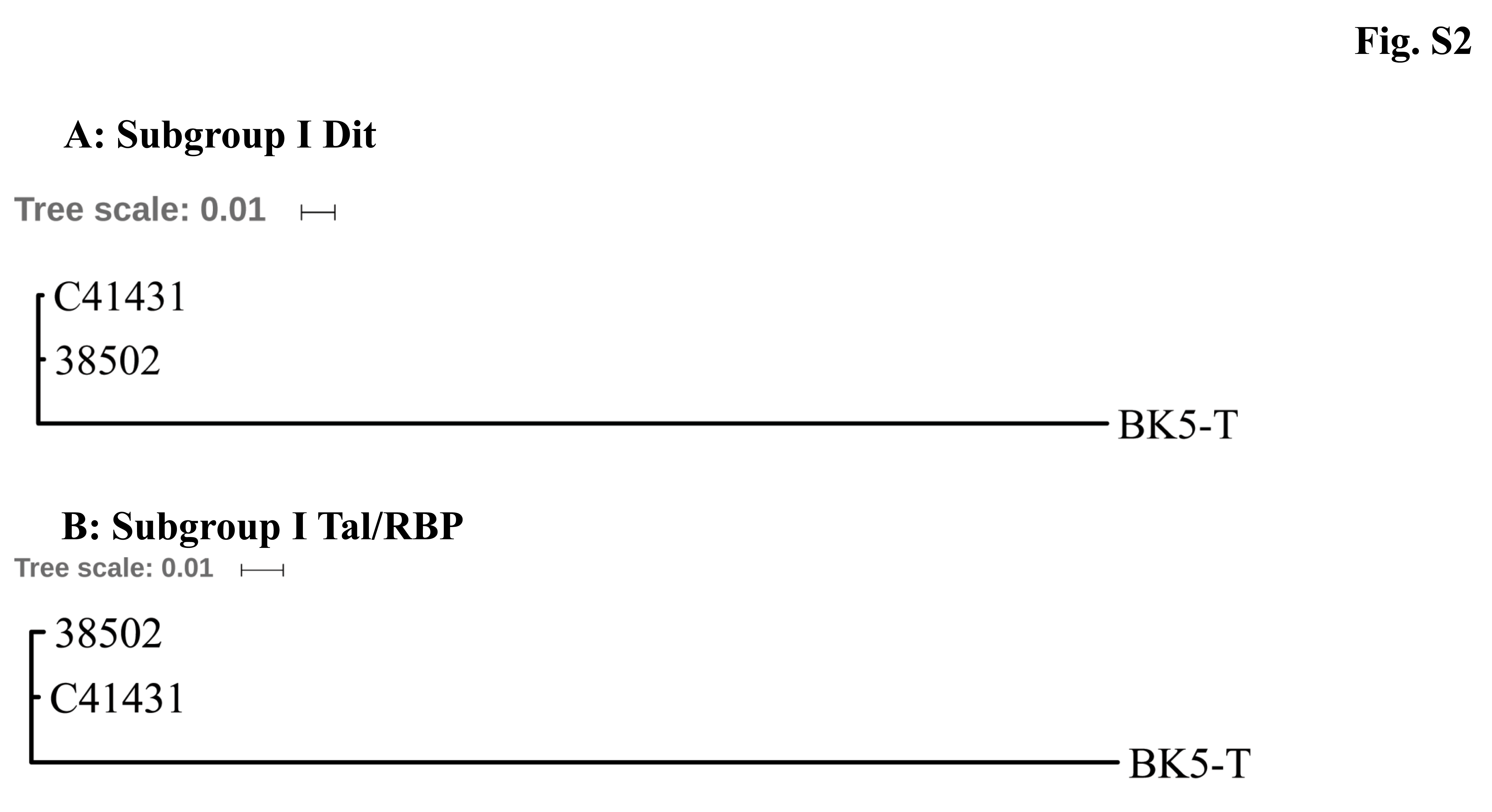

Supplement: Additional file 2: Figure S2. — Unrooted phylogenetic tree of the Dit (Panel A) and Tal/RBP (Panel B) protein sequences of the phages possessing a sub-group I RBP: BK5-T, 38502 and C41431. (TIFF 505 kb) [file 12864_2017_3537_MOESM2_ESM.tiff]

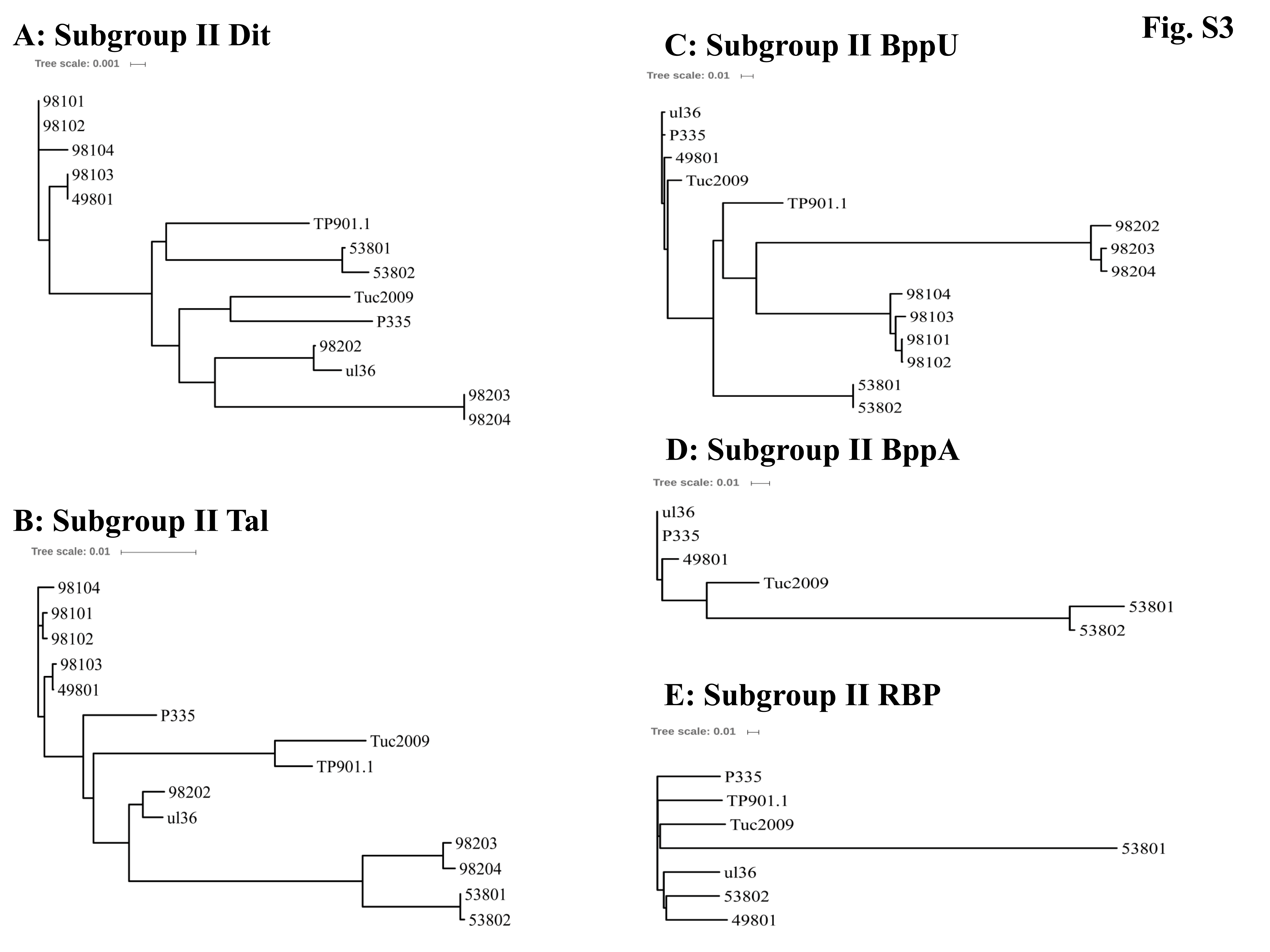

Supplement: Additional file 3: Figure S3. — Unrooted phylogenetic tree of the Dit (Panel A), Tal (Panel B), BppU (Panel C), BppA (Panel D) and RBP (Panel E) proteins of the sub-group II phages. (TIF 1257 kb) [file 12864_2017_3537_MOESM3_ESM.tif]

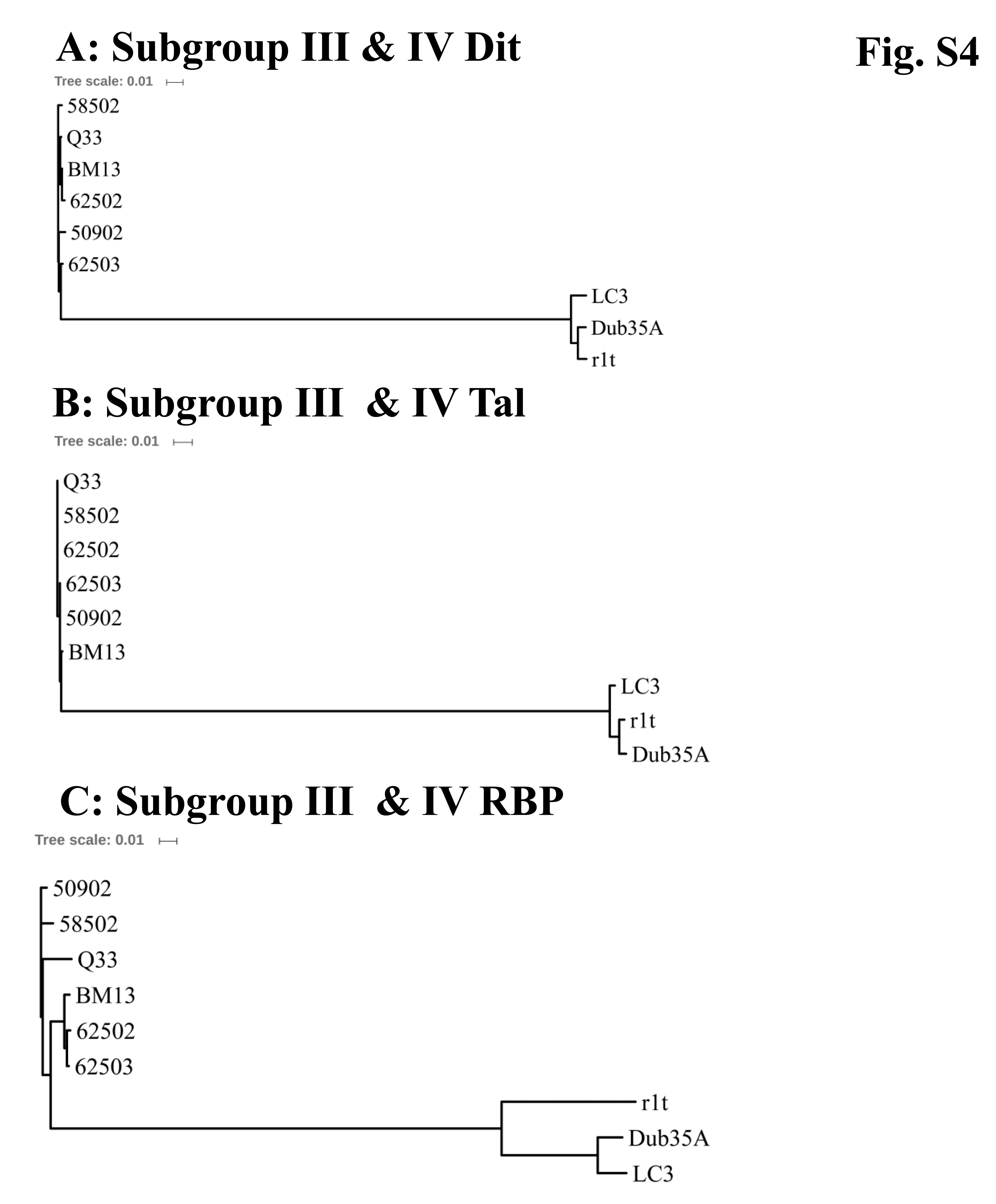

Supplement: Additional file 4: Figure S4. — Unrooted phylogenetic tree of the Dit (Panel A), Tal (Panel B) and RBP (Panel C) proteins of the sub-group III and sub-group IV phages. (TIFF 593 kb) [file 12864_2017_3537_MOESM4_ESM.tiff]

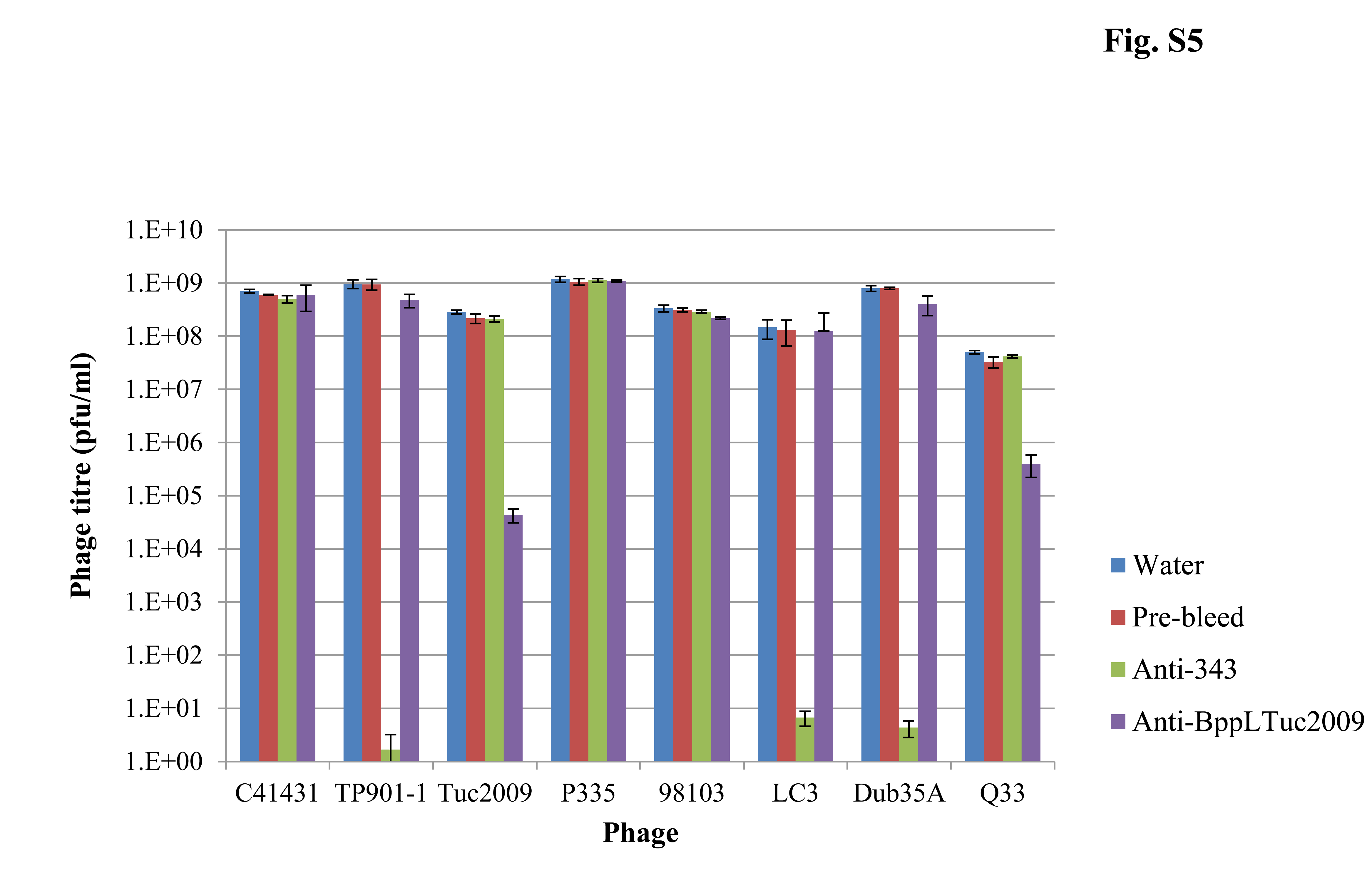

Supplement: Additional file 5: Figure S5. — Bar chart representing the titre of phages tested in antibody neutralisation assays. The blue bar represents a control in which water was included in place of antibodies to assess the control titre; the red bars represent a control in which pre-bleed serum was added in place of the antibodies to reflect the effect of serum on the phages; the green bars represent the effect of incubation of anti-343LC3 on the plaquing ability of the assessed phages and; the purple bars represent the effect of incubation of anti-BppLTuc2009 on the plaquing ability of the tested phages. Representatives of each of the four P335 phage sub-groups were selected for this analysis with C41431 used as the representative of the sub-group I since its predicted RBP bears homology to that of the sub-group I phage BK5-T. All data represented are the average of at least three independent assays. (TIFF 453 kb) [file 12864_2017_3537_MOESM5_ESM.tiff]
